# Inflammatory Mediators of Axon Regeneration in the Central and Peripheral Nervous Systems

**DOI:** 10.3390/ijms242015359

**Published:** 2023-10-19

**Authors:** Larry I. Benowitz, Lili Xie, Yuqin Yin

**Affiliations:** 1 Department of Neurosurgery, Boston Children’s Hospital, Boston, MA 02115, USA; lili.xie@csu.edu.cn (L.X.); yuqin.yin@childrens.harvard.edu (Y.Y.); 2 F.M. Kirby Neurobiology Center, Boston Children’s Hospital, Boston, MA 02115, USA; 3 Department of Neurosurgery, Harvard Medical School, Boston, MA 02115, USA; 4 Department of Ophthalmology, Harvard Medical School, Boston, MA 02115, USA; 5 Department of Ophthalmology, University of Pittsburgh Medical Center, Pittsburgh, PA 15213, USA; 6 Department of Ophthalmology, Second Xiangya Hospital, Central South University, Changsha 410011, China

**Keywords:** inflammatory cells, growth factors, axon regeneration, oncomodulin, SDF1, CCL5, ArmC10, conditioning lesion

## Abstract

Although most pathways in the mature central nervous system cannot regenerate when injured, research beginning in the late 20th century has led to discoveries that may help reverse this situation. Here, we highlight research in recent years from our laboratory identifying oncomodulin (Ocm), stromal cell-derived factor (SDF)-1, and chemokine CCL5 as growth factors expressed by cells of the innate immune system that promote axon regeneration in the injured optic nerve and elsewhere in the central and peripheral nervous systems. We also review the role of ArmC10, a newly discovered Ocm receptor, in mediating many of these effects, and the synergy between inflammation-derived growth factors and complementary strategies to promote regeneration, including deleting genes encoding cell-intrinsic suppressors of axon growth, manipulating transcription factors that suppress or promote the expression of growth-related genes, and manipulating cell-extrinsic suppressors of axon growth. In some cases, combinatorial strategies have led to unprecedented levels of nerve regeneration. The identification of some similar mechanisms in human neurons offers hope that key discoveries made in animal models may eventually lead to treatments to improve outcomes after neurological damage in patients.

## 1. Historical Background

The inability of neurons to regenerate injured axons within the central nervous system (CNS) has dire consequences for victims of spinal cord injury, stroke, optic nerve damage, and multiple neurodegenerative diseases. Even in the peripheral nervous system (PNS), in which axon regeneration occurs spontaneously, it is often incomplete, leaving many patients with residual deficits. Here, we focus primarily on the role of immune-derived factors in promoting axon regeneration in the optic nerve, an area in which our laboratory has worked for many years, but also include mention of related research from other laboratories and our research on the role of immune-derived factors in promoting axon regeneration in peripheral nerves and spinal cord. 

Like other CNS pathways, the optic nerve normally exhibits no ability to regenerate after injury. However, in 1911, Tello and Ramón y Cajal reported the first evidence that mature retinal ganglion cells (RGCs), the projection neurons of the retina, retain a capacity to regenerate injured axons when presented with an autologous sciatic nerve graft affixed to the cut end of the optic nerve [1]. Aguayo and colleagues later extended these findings [2], showing that, under certain circumstances, mature RGCs can regenerate axons the full length of a peripheral nerve graft and form synapses in the superior colliculus, the principal target of RGCs in mice and rats [3]. 

In view of the many studies at the time demonstrating the inhibitory influences of CNS myelin on axon growth [4,5,6,7], the findings of Cajal’s and Aguayo’s groups were widely interpreted as resulting from the permissive glial environment of the PNS contrasted with the suppressive glial environment of the CNS. However, in 1996, Martin Berry reported that RGCs could regenerate axons into the optic nerve itself when a fragment of a previously injured peripheral nerve was placed into the vitreous chamber of the eye [8]. These experiments were first taken to suggest that trophic factors secreted by Schwann cells were an underappreciated factor in the peripheral nerve graft experiments, a hypothesis that was supported by controls showing that freezing and thawing the graft diminished regeneration. However, the intraocular grafts of Berry’s studies contained numerous inflammatory cells, which subsequent studies suggest are the actual enablers of optic nerve regeneration.

## 2. Intraocular Inflammation Enables Optic Nerve Regeneration: Role of Oncomodulin

**Figure 1:**
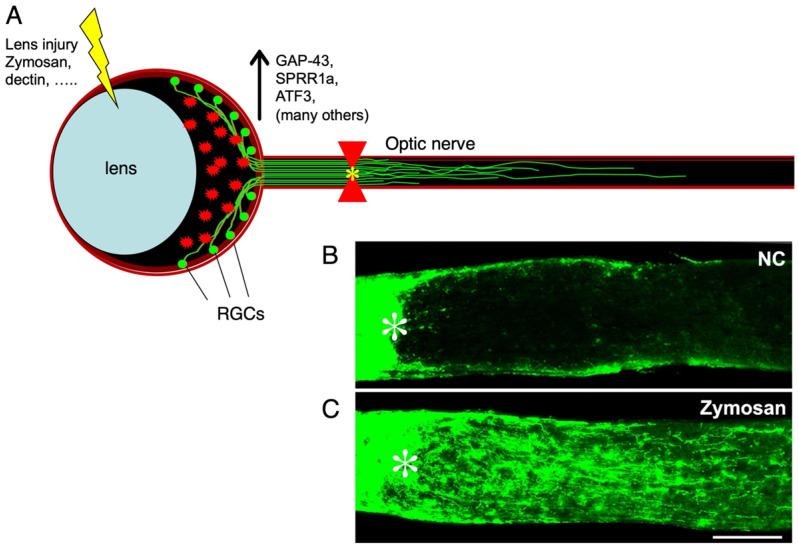
Intraocular inflammation promotes optic nerve regeneration. (**A**) schematic illustration showing the effects of intraocular inflammation (via lens injury or intravitreal zymosan or dectin) in causing retinal ganglion cells (RGCs) to increase expression of growth-associated genes (e.g., GAP-43, SPRR1a, ATF3) and regenerate injured axons down the optic nerve beyond the injury site (white asterisk). (**B**,**C**) longitudinal optic nerve sections immunostained for the growth-associated protein GAP-43 (green). NC: nerve crush only. Scale bar in (**C**), 200 µm. Zymosan-induced inflammation stimulates RGCs to regenerate axons well beyond the nerve injury site (asterisk). (from refs. [9,11,13]; Panel (**B**) reprinted with permission from ref. [17]. Copyright 2022 Springer Nature).

In 2000, we discovered that intraocular injections that encroached upon the lens were sufficient to induce considerable levels of optic nerve regeneration (Figure 1) [9] (also [10]). Lens injury leads to a massive influx of inflammatory cells, and its effects on regeneration can be mimicked by the pro-inflammatory agent zymosan, a yeast cell wall preparation [11], recalling earlier reports on the possible pro-regenerative effects of activated macrophages injected into the injured optic nerve [12]. At the transcriptional level, intraocular inflammation was found to induce massive changes in RGCs’ program of gene expression, resembling those seen in primary sensory neurons undergoing regeneration of their peripheral axons, and partially recapitulating the transcriptional program associated with the initial development of neural connections [13]. The effects of zymosan in inducing regeneration are mimicked or exceeded using defined Toll-like receptor ligands or injecting an immature population of neutrophils [14,15,16]. 

**Figure 2:**
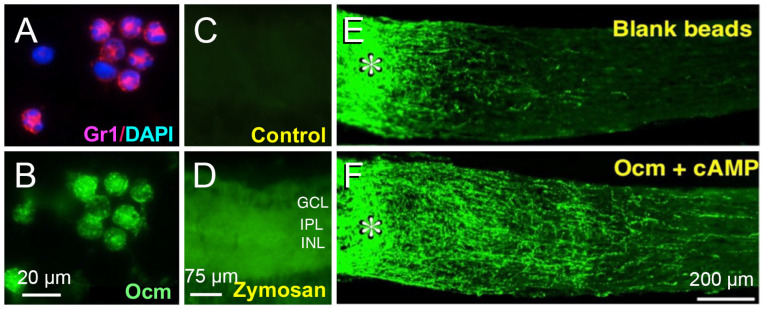
Inflammatory cell-derived oncomodulin (Ocm) promotes optic nerve regeneration. (**A**,**B**) intravitreal injection of zymosan leads to infiltration of neutrophils (red in (**A**), Gr1 immunostaining) expressing Ocm (green in (**B**)). (**C**,**D**) Ocm immunostaining in the inner retina following intravitreal zymosan injection (GCL: ganglion cell layer; IPL: inner plexiform layer; INL: inner nuclear layer). (**E**,**F**) slow-release microspheres loaded with Ocm and CPT-cAMP (Ocm+cAMP) injected into the vitreous induce optic nerve regeneration. Asterisks: injury site. (from refs. [18,22]. Panels (**E**,**F**) reprinted with permission from ref. [18]. Copyright 2006, Springer Nature).

The effects of intraocular inflammation on optic nerve regeneration were not mimicked by a host of well-characterized trophic factors (e.g., BDNF, CNTF, FGF) [9,11,18], necessitating a search for novel immune-derived trophic factors. We discovered that monocytes in culture, when stimulated with zymosan, secrete a protein < 30 kDa that, in the presence of a cAMP analog and the carbohydrate mannose, enabled RGCs to extend lengthy axons [11,18]. Further purification and analysis by mass spectrometry identified the active protein as oncomodulin (Ocm), a small calcium-binding protein previously associated only with particular hair cells of the inner ear [19,20,21]. Nonetheless, Ocm was found to be highly expressed by infiltrative neutrophils, the first responders of the innate immune system, and to a lesser extent in infiltrative macrophages. Levels of Ocm mRNA and protein increase dramatically in the eye following intraocular inflammation, and gain-of-function experiments showed that Ocm, when packaged together with a cAMP analog in slow release polymer beads, mimicked the pro-regenerative effects of zymosan-induced inflammation (Figure 2) [18]. Conversely, immune depletion of neutrophils or a peptide antagonist of Ocm greatly diminished the effects of intraocular inflammation on optic nerve regeneration [22,23]. 

## 3. SDF1 Complements the Pro-Regenerative Effects of Ocm

**Figure 3:**
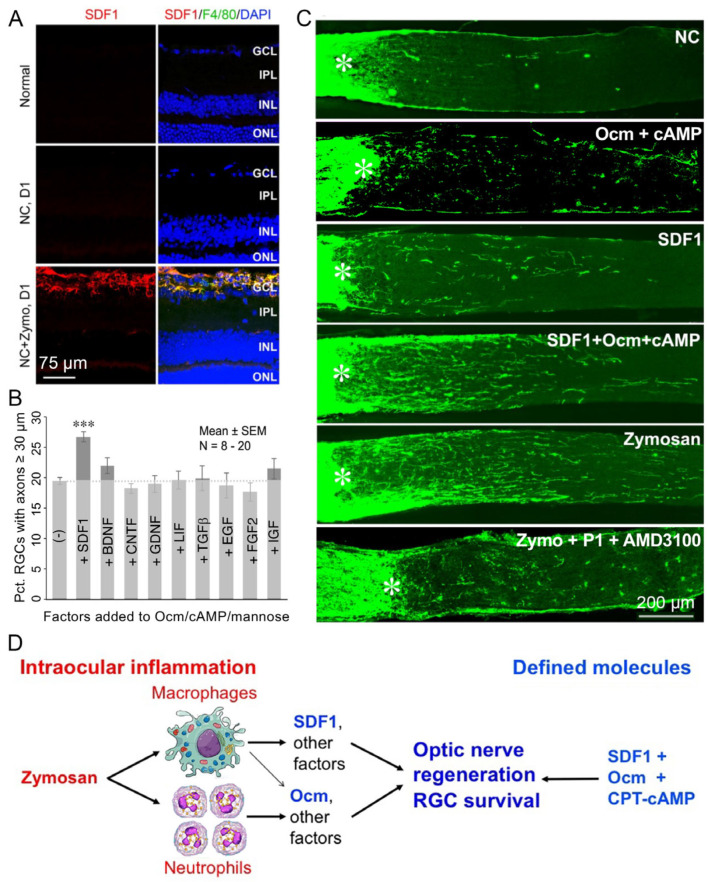
Macrophage-derived SDF1 stimulates optic nerve regeneration. (**A**) one day after zymosan intraocular injection, macrophages (green, F4/80 immunostaining) expressing SDF1 (red) infiltrate into the retina. (**B**) in adult rat RGC cultures, SDF1 is the only factor among many known growth factors tested that augments the effects of Ocm and its co-factors cAMP and mannose. *** *p* < 0.001 compared to Ocm/cAMP/mannose alone. (**C**) in vivo, a single intravitreal injection of SDF1 has mild effects on promoting axon growth on its own and augments regenerative growth when combined with Ocm+cAMP. Specific inhibitors to SDF1 (AMD3100) and Ocm (P1 peptide) nearly eliminate regeneration induced by intraocular inflammation. NC: nerve crush only. (**D**) schematic summary of effects of inflammatory cell-derived growth factors. After zymosan injection in vitreous, infiltrative macrophages and neutrophils express Ocm, SDF1, and other factors, and stimulate optic nerve regeneration and RGC protection. Use of the defined molecules mimics these effects (from ref. [24]). Panels (**A**–**C**) reproduced with permission from ref. [24], copyright 2022, National Academy of Sciences.

Although our experiments showed that Ocm is essential for inflammation-induced regeneration, further studies pointed to the existence of important complementary factors. Thus, whereas slow-release polymer beads releasing recombinant Ocm (rOcm) plus a cAMP analog mimicked the pro-regenerative effects of zymosan, we later discovered that a single intravitreal injection of rOcm with a cAMP analog had little effect on its own (Figure 3). This finding implied that the beads per se may have contributed to the earlier result, as suggested by the low level of outgrowth induced by injecting blank beads alone [18]. Although none of the well-characterized factors that we tested mimicked the effects of Ocm, we discovered that the chemokine SDF1 (CXCL12) exhibits some pro-regenerative and neuroprotective effects on its own and strongly augmented the effects of Ocm [24] (Figure 3). SDF1 is highly expressed in infiltrative monocytes following intraocular zymosan injections (Figure 3A), and deleting the gene encoding SDF1 in monocytes (LysM-Cre: *Cxcl12*^flx/flx^ mice) or injecting an SDF1 antagonist (AMD3100) intraocularly strongly suppressed zymosan-induced regeneration. Conversely, combining recombinant SDF1 with rOcm and a cAMP analog mimicked or exceeded the effects of intraocular inflammation [24] (Figure 3), indicating that the beneficial effects of intraocular inflammation, itself not a viable clinical strategy, can be attained with defined factors. 

Other work has shown that the chemokine CXCL5 and receptor CXCR2 also promote optic nerve regeneration, whereas an antagonist to these chemokines suppresses the pro-regenerative effects of other inflammatory stimuli [25]. Finally, a large-scale screen identified interleukin IL-22 as a suppressor of optic nerve regeneration [26]. 

## 4. CNTF Gene Therapy Induces Optic Nerve Regeneration Indirectly via CCL5

**Figure 4:**
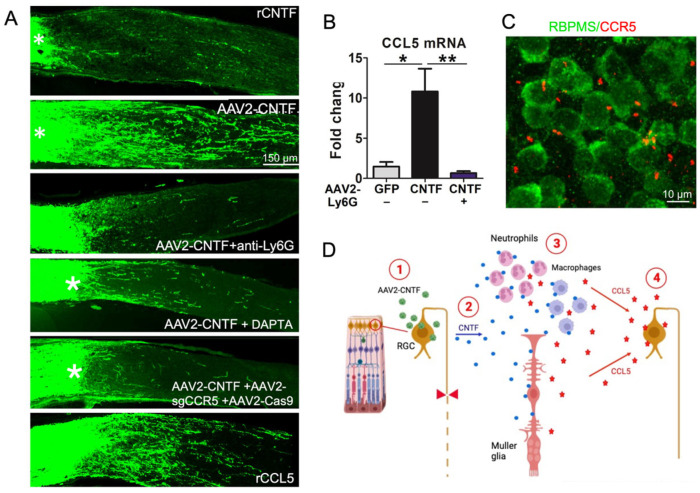
The effects of CNTF gene therapy are mediated by immune cell-derived CCL5. (**A**) longitudinal sections through the injured mouse optic nerve showing regenerating GAP-43-positive axons after different treatments. Whereas recombinant CNTF has little effect, CNTF gene therapy (AAV2-CNTF) induces robust regeneration (first two panels). Immune depletion of neutrophils abrogates the effect of CNTF gene therapy (third panel), as does DAPTA, an antagonist of the CCL5 receptor CCR5 and CCR5 deletion in RGCs via CRISPR-Cas9 and a small gRNA directed against CCR5 (panels 4 and 5). Recombinant CCL5 induces appreciable regeneration (panel 6). Asterisks denote injury site. (**B**) CNTF gene therapy elevates expression of CCL5 in the retina, an effect that is eliminated by immune depletion of neutrophils. * *p* < 0.05 (effect of CNTF gene therapy compared to control virus); ** *p* < 0.01 (effect of CNTF gene therapy without vs. with neutrophil depletion). (**C**) the CCL5 receptor CCR5 is expressed on cilia that extend from RBPMS-positive RGCs. (**D**) schematic illustration showing the role of CCL5 in mediating the effects of CNTF gene therapy. AAV2-CNTF infection of RGCs (1) induces inflammation that is amplified by CNTF expression (2). CNTF induces inflammatory cells and retinal Mueller cells to express chemokine CCL5 (3), which acts directly on RGCs to promote axon regeneration (4). Panels (**A**–**C**) reprinted with permission from ref. [41] Copyright 2021, National Academy of Sciences.

Another growth factor that has been widely used to promote optic nerve regeneration is ciliary neurotrophic factor (CNTF). Surprisingly, however, although CNTF gene therapy has been shown repeatedly to be highly effective [27,28,29,30,31,32,33,34] in this regard, CNTF delivered intraocularly as a recombinant protein at physiological levels has only minor effects [9,35,36]. After confirming this discrepancy (Figure 4A), we went on to investigate the underlying causes. Previous studies had shown that intraocular delivery of adeno-associated viruses promotes inflammation [37] and that CNTF can act as a chemoattractant for inflammatory cells [38,39]. Together, these studies suggested that perhaps CNTF gene therapy works by amplifying the pro-inflammatory effects of intraocular virus delivery and elevating levels of other immune cell-derived growth factors. This idea was confirmed by showing that neutrophil depletion abrogates the effects of CNTF gene therapy (Figure 4A). An earlier study had shown that CNTF elevates expression of the chemokine CCL5 in Mueller cells [40]. We confirmed that CNTF gene therapy elevates CCL5 expression in the retina and further discovered that RGCs express the cognate receptor, CCR5, on their cilia (Figure 4) [41]. Deletion of CCR5 in RGCs via CRISPR- Cas9 or the CCR5 antagonist DAPTA nearly eliminated the effects of *Cntf* gene therapy (Figure 4A, panels 4 and 5), and conversely, recombinant CCL5 mimicked the effects of *Cntf* gene therapy (Figure 4, panel 6). In sum, then, CCL5 mediates most, if not all, of the beneficial effects of *Cntf* gene therapy (Figure 4C), and thus CCL5 joins Ocm and SDF1 as immune cell-derived factors that promote optic nerve regeneration [41]. 

## 5. A Conditioning Effect in Optic Nerve Regeneration

**Figure 5:**
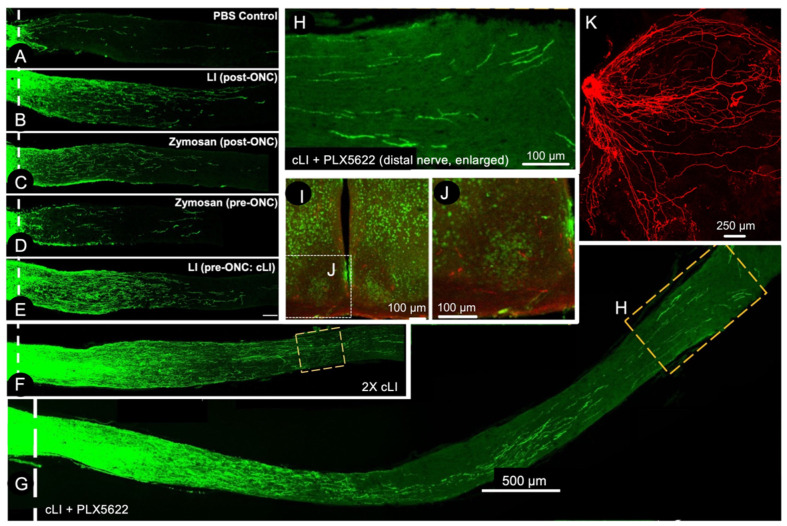
Conditioning by lens injury induces robust optic nerve regeneration. (**A**–**G**) longitudinal sections through the mouse optic nerve showing GAP-43-positive regenerating axons following various treatments. (**A**–**C**) whereas saline-treated control case shows little axon regeneration after optic nerve injury, lens injury (LI), or zymosan administered shortly after nerve injury induce modest levels of regeneration. (**D**,**E**) whereas zymosan treatment two weeks prior to nerve crush has little or no effect; a small puncture of the lens capsule at the same time point has much stronger effects. (**F**) two episodes of lens injury two weeks and one week prior to nerve crush more than doubles the effect of a single conditioning injury. (**G**,**H**) multiple episodes of lens injury combined with microglial inhibition (using PLX5622) enables some RCS to regenerate axons the full length of the optic nerve in just two weeks. (**I**,**J**) this same combinatorial treatment enables some RGCs to regenerate CTB-labeled axons (red) into central target areas, including the suprachiasmatic nucleus. (**K**) βIII tubulin-positive axons that arise from RGCs regenerating over the surface of the lens (Reprinted with permission from ref. [50] Copyright 2023, Journal of Clinical Investigation).

In the peripheral nervous system, an initial injury to the sciatic nerve primes injured sensory neurons of the dorsal root ganglia to show accelerated regeneration upon a second injury [42,43,44,45]. This phenomenon, called the conditioning lesion effect, is mediated by infiltrative monocytes that secrete oncomodulin and perhaps other factors [42,46,47,48,49]. We now find that inflammatory conditioning also has profound effects on optic nerve regeneration [50]. As noted earlier, either lens injury or zymosan at the time of optic nerve crush leads to moderate levels of optic nerve regeneration. However, if a mild injury restricted to the lens capsule is performed two weeks prior to optic nerve crush, the resulting level of regeneration is several-fold higher than seen with either zymosan or lens injury at the time of nerve crush (Figure 5), and zymosan delivery two weeks prior to optic nerve crush has no benefit [50]. Repeated episodes of lens injury pre- and post-optic nerve crush result in even greater levels of regeneration, enabling RGCs to regenerate axons the full length of the optic nerve and into the optic chiasm within 3–4 weeks [50]. Blocking neutrophils had no effect on this phenomenon, while blocking monocyte entry partially diminished the effect, and suppressing microglial proliferation using the Csf-1 receptor inhibitor PLX5622 doubled the level of regeneration attained with multiple episodes of lens-injury conditioning, enabling some RGC axons to enter the suprachiasmatic and lateral geniculate nuclei after just a few weeks (Figure 5). Blocking Ocm, SDF1, and CCL5 also failed to diminish the effects of conditioning lens injury, raising the question of what factors enable this striking phenomenon [50].

Inflammation also contributes to regeneration through clearance of myelin debris. In the mouse optic nerve injury model, deleting genes encoding key elements of the classical complement cascade (complement proteins C1q and C3 or the complement receptor CR3) diminishes regeneration induced by several means. Myelin debris accumulates in active microglia that appear to be infiltrative in origin, and is likely to represent the mechanism by which inflammation supports axon regeneration. However, when regeneration was stimulated by the strong combinatorial therapy described below, regeneration proceeded at a near-normal pace despite inactivation of the classical complement cascade and correlates with down-regulation of the Nogo receptor NgR, a receptor for several myelin-inhibitory and scar-related proteins [51]. 

## 6. Combinatorial Approaches

Following the discovery that CNS myelin suppresses axon outgrowth, several groups went on to characterize the molecular bases of this inhibition, identifying Nogo A, B, and C, myelin associated glycoprotein (MAG), and oligodendrocyte-myelin glycoprotein (OMgp) [7,52,53,54], along with their receptors and intracellular proteins that transduce the effects of inhibitory molecules, as the major players [55,56,57,58]. In addition, the fibrotic scar that forms at the site of nerve injury is also highly suppressive to axon regeneration, a phenomenon associated primarily with sulfated proteoglycans [59,60,61,62,63,64,65,66,67,68,69,70,71,72,73]. Nonetheless, methods to counteract these inhibitory factors, including genetic deletion of genes encoding receptors for the inhibitory proteins or overexpression of a dominant negative form of the receptor that mediates the effects of these proteins, leads to only modest or no axon regeneration in the optic nerve or spinal cord [13,74,75,76,77,78,79]. However, combining methods to counteract cell-extrinsic suppressors of axon growth with intraocular inflammation to activate RGC’s intrinsic growth program results in far greater optic nerve regeneration than occurs after intraocular inflammation alone [13,76,77,78].

**Figure 6:**
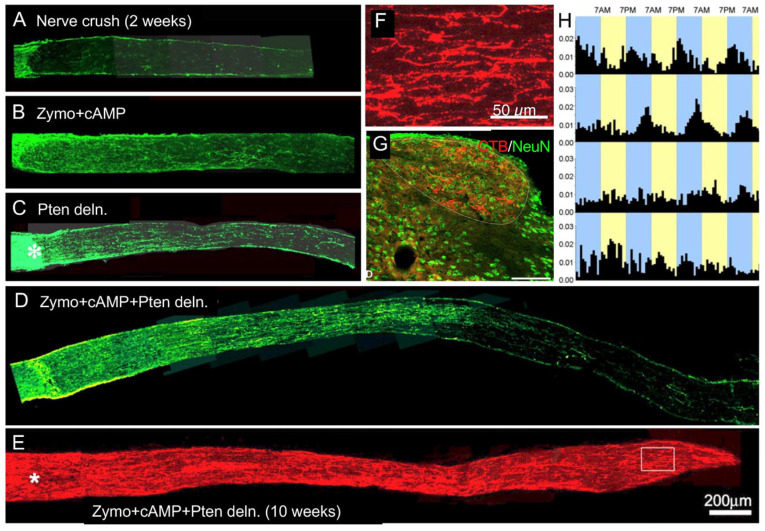
Synergistic effects of intraocular inflammation and Pten gene deletion. (**A**–**C**) intraocular inflammation plus the cAMP analog CPT-cAMP and PTEN deletion in RGCs each induces appreciable levels of regeneration. (**D**) combining these treatments has strongly synergistic effects, enabling some RGCs to regenerate axons half the length of the optic nerve in just two weeks. (**E**–**G**) continuing this same treatment for 10 weeks enables many RGCs to extend axons the full length of the optic nerve (**E**); enlarged area near the chiasm shown in (**F**) and into central visual relay areas, including the dorsal lateral geniculate nucleus (**G**; scale bar, 100 µm). (**H**) average circadian activity over the course of several days in groups of normal mice (top row) and in mice with optic nerve damage receiving the combined treatment (Zymosan/cAMP/Pten deletion, second row), control treatment (omitting Pten deletion, third row), or no treatment (i.e., blind mice, fourth row). Whereas mice with the full treatment show synchronous circadian activity, albeit phase-shifted relative to normal animals, mice with partial treatment and blind mice are out of phase with each other and thus show less coherent group activity. (from refs. [81,82]). Asterisks denote injury site. Panels (**A**–**D**) are used with permission from ref. [82] (Copyright 2010, Society for Neuroscience); panels (**E**–**H**) reprinted with permission from ref. [81] (Copyright 2012, National Academy of Sciences).

A potent alternative way to activate RGCs’ intrinsic growth program and induce regeneration is to counteract cell-intrinsic suppressors of growth. In many cell types, PTEN suppresses the PI3K-AKT pathway which controls growth at the transcriptional and translational levels. In RGCs, *Pten* gene deletion is sufficient to induce appreciable levels of optic nerve regeneration [80] (Figure 6), and combining intraocular inflammation (via zymosan) with *Pten* deletion enables some RGCs to regenerate axons the full length of the optic nerve, across the optic chiasm, and into appropriate target areas within 10 weeks, with partial recovery of simple visually guided behaviors [81] (Figure 6).

The protein SOCS3 suppresses signaling through the JAK-STAT pathway, and deleting the *Socs3* gene in RGCs induces some regeneration on its own and enables RGCs to respond to CNTF [35]. Combining *Pten* and *Socs3* deletion with CNTF represents another method to induce long-distance axon regeneration [83], although within the optic chiasm and beyond, axons appear unable to navigate to appropriate targets, an issue that may also be true of many axons induced to regenerate in response to intraocular inflammation combined with PTEN deletion [84].

## 7. Differential Responses of RGC Subtypes

**Figure 7:**
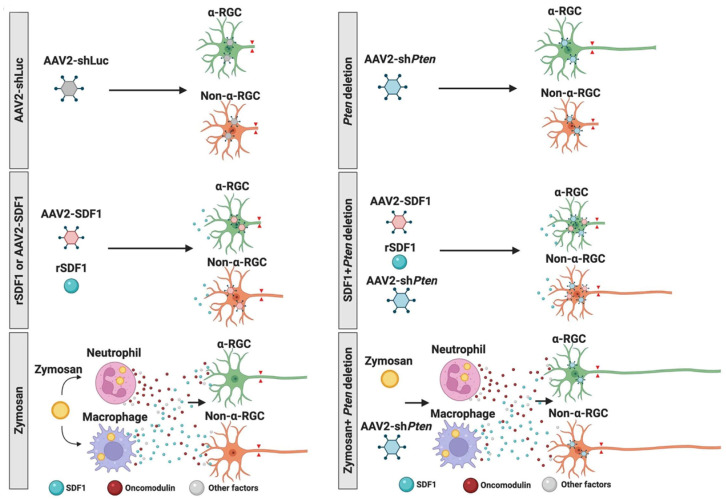
Responsiveness of RGC subtypes to Pten deletion, SDF1, and intraocular inflammation. **Top panel:** whereas a control AAV2 virus expressing an shRNA to luciferase (AAV2-shLuc) does not induce any axon growth (**left**), AAV2-*shPten* preferentially causes αRGCs to regenerate axons (**right**). (**Middle panel**): SDF1, either encoded by a virus or a recombinant protein (AAV2-SDF1, rSDF1), stimulates non-αRGCs to regenerate axons (**left**), and this selectivity is maintained even with the addition of AAV2-*shPten* (**right**). Bottom panel: Zymosan-induced regeneration is from both α- and non- αRGCs (**left**), and the addition of AAV2-*shPten* augments regeneration from both (**right**). (reprinted with permission from ref. [24], Copyright 2022, National Academy of Sciences).

RGCs have long been known to represent a heterogeneous cell population in terms of size, dendritic branching patterns, molecular composition, connectivity, and physiological properties [85,86,87,88]. With the advent of single-cell sequencing, over 40 distinct RGC subtypes have been identified in mice [89,90]. Many fewer subtypes are found in humans and non-human primates, with one type, midget RGCs, dominating [91]. In mice, deletion of *Pten* in RGCs favors the survival and regenerative potential of α-RGCs [92], whereas SDF, in contrast, selectively induces axon regeneration from non-α-RGCs. Combining SDF1, Ocm, and PTEN deletion synergistically enhances the regeneration of axons from both α- and non-α-RGCs [24] (Figure 7). 

A recent bioinformatic study has delved deeper into the question of how different RGC subtypes respond to various treatments. By combining retrograde labeling to distinguish RGCs that do or do not successfully regenerate axons with single-cell sequencing, Jacobi et al. confirmed that *Pten* deletion favors the regeneration of αRGCs, and found that SOCS3 deletion or combining Pten deletion and SOCS3 deletion each favors axon regeneration in distinct RCC subtypes. Importantly, combining Pten and Socs3 deletion with CNTF enabled a wide spectrum of RGC subtypes to regenerate axons [93].

## 8. ArmC10, the Ocm Receptor, Is Critical for Inflammation-Induced Optic Nerve Regeneration and the Conditioning Lesion Effect in the Peripheral Nervous System

**Figure 8:**
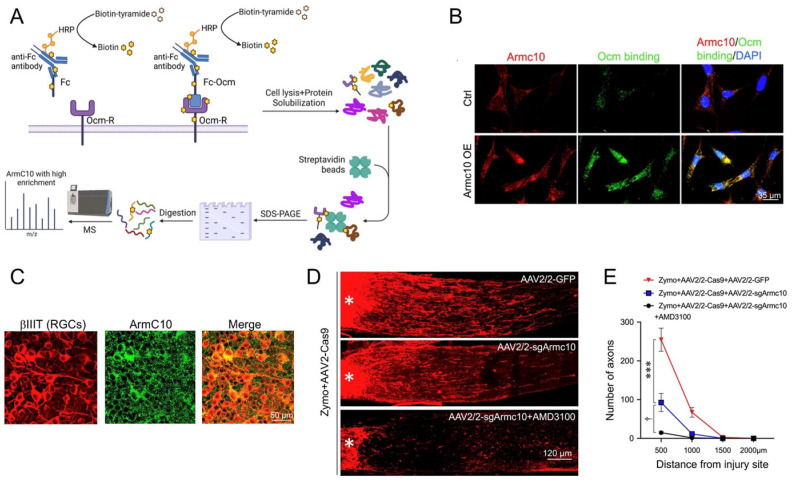
Identification of ArmC10 as a high-affinity oncomodulin receptor. (**A**) identification of a candidate receptor. Using a variant form of proximity biotinylation, lightly fixed RGCs in culture were incubated with either an Fc control probe or an Fc-Ocm fusion protein (“bait”), rinsed, and then exposed to an anti-Fc antibody conjugated to the oxidizing enzyme horseradish peroxidase (HRP). Biotinylation was carried out using the biotin donor (biotin tyramide) in the presence of H_2_O_2_. Proteins were extracted from lysed cells and biotinylated proteins were adsorbed onto streptavidin beads that were then separated by SDS-PAGE. Following enzymatic digestion of proteins, peptides were analyzed by mass spectrometry. After excluding obvious impurities, ArmC10 emerged as the protein with the highest enrichment in samples probed with Fc-Ocm vs. Fc. (**B**) gain-of-function studies. After identifying a cell line with low endogenous ArmC10 expression, cells were transfected with a control plasmid or a plasmid encoding ArmC10, then incubated with recombinant Ocm and immunostained to visualize levels of ArmC10 and Ocm. Note elevated Ocm binding following transfection with the ArmC10 plasmid. (**C**) endogenous ArmC10 expression in whole-mounted retina immunostained for βIII tubulin (to visualize RGCs) and ArmC10. (**D**) loss-of-function studies. Virally mediated deletion of ArmC10 in RGCs (intraocular AAV2-sgArmC10 + AAV2-Cas9) diminishes axon regeneration induced by Zymosan + CPT-cAMP. (**E**) addition of the SDF1 antagonist AMD3100 eliminates Zymosan-induced regeneration almost completely. ^†^ *p* < 0.05; *** *p* < 0.001. (Reprinted with permission from ref. [49]. Copyright 2023, American Academy of Arts and Sciences).

Despite its prominent role in inflammation-induced optic nerve regeneration, the mechanisms by which Ocm exerts its effects had long remained a mystery. Using a modification of the biotinylation by antibody recognition (BAR) method [94], we identified ArmC10 as a candidate Ocm receptor and validated its role through co-immunopre-cipitation, direct measurement of binding affinity, and by gain-of-function and loss-of-function studies (Figure 8A,B). ArmC10 bears no homology to other known growth factor receptors, nor are other members of the Arm repeat family cell surface receptors. Nonetheless, RGCs were found to express high levels of ArmC10 on their cell surface, and RGC-selective *ArmC10* deletion greatly diminished the effects of intraocular inflammation in promoting optic nerve regeneration [49] (Figure 8D,E) [49]. 

As noted above, infiltrative monocytes and Ocm contribute to the conditioning lesion effect in the peripheral nervous system [46,95,96]. In cell culture, Ocm stimulates mouse sensory neurons to extend neurites [18,46], and this effect is lost upon ArmC10 deletion [49]. In vivo, Ocm gene therapy enhances the regeneration of peripheral nerve axons and promotes regeneration of ascending sensory axons in the dorsal columns of the spinal cord. In contrast, ArmC10 deletion in sensory neurons diminishes the effects of a conditioning lesion on both peripheral nerve regeneration and the regeneration of ascending sensory axons in the injured spinal cord [49]. 

## 9. SDF1 Complements the Effects of Ocm on Peripheral Sensory Neurons

**Figure 9:**
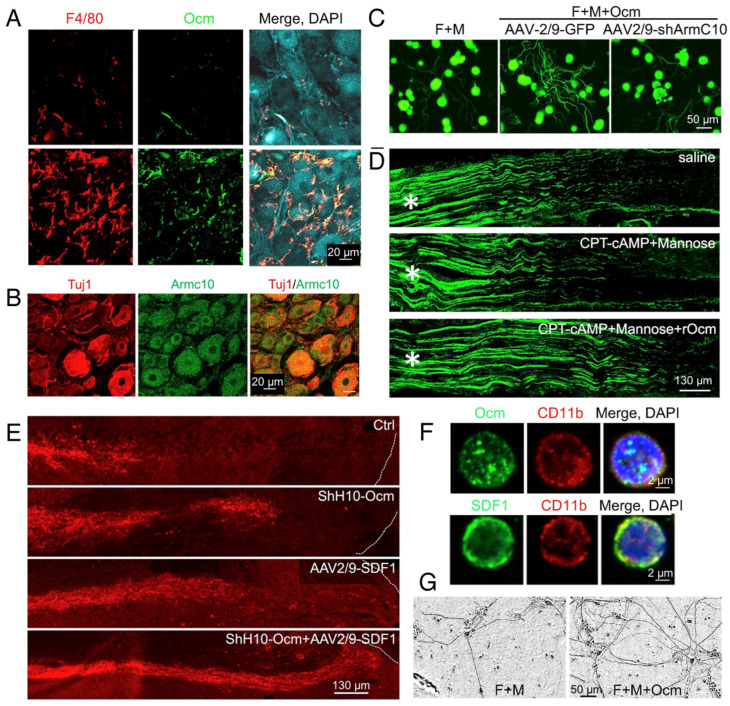
Role of Ocm, ArmC10 and SDF1 in axon regeneration in the sciatic nerve and spinal cord. (**A**) upper row: Sections through normal adult mouse dorsal root ganglion (DRG) show little monocyte infiltration (F4/80 immunostaining, red) or Ocm (green). Large nuclei of principle sensory neurons are visualized in the last panel with DAPI (blue). Lower row: peripheral nerve injury causes an influx of F4/80-positive monocytes that express Ocm into the DRG. (**B**) sensory neurons (stained with antibody TUJ1 for βIII tubulin, red) express the Ocm receptor ArmC1 (green). (**C**) neurite outgrowth from DRG sensory neurons in culture. Treatment with forskolin, mannose, and Ocm (F + M + Ocm) induces neurite outgrowth in controls infected with AAV2/9 carrying the GFP gene but not in cells infected with the ArmC10 knock-down virus AAV2/9-shArmC10. (**D**) Ocm with co-factors (CPT-AMP, mannose) accelerates axon regeneration following sciatic nerve injury (injury site marked at left with an asterisk). (**E**) longitudinal sections through adult mouse spinal cords following thoracic injury (right side of images) and the indicated treatments. In untreated controls, ascending sensory axons (red) retract from the injury site and do not regenerate back (top panel). Infection with viruses expressing either Ocm (ShH10-Ocm) or SDF1 (AAV2/9-SDF1) enables axon regeneration half way back to the injury site (panels 2 and 3), whereas overexpression of both Ocm and SDF1 enables axons to regenerate all the way back to the injury site (bottom panel). (**F**) expression of Ocm (upper row) and SDF1 (lower row) in human blood-derived monocytes (immunstained with antibody to CD11b). (**G**) Ocm stimulates neurite outgrowth from human pluripotent stem cells induced to differentiate into peripheral sensory neurons. Note increased growth with the addition of Ocm to forskolin + mannose. Reprinted with permission from ref. [49]. Copyright 2023, American Academy of Arts and Sciences.

As in the optic nerve, the effects of Ocm on peripheral sensory neurons are strengthened by SDF1. In addition to expressing ArmC10, sensory neurons express the SDF1 receptor CXCR4, and deleting CXCR4 in sensory neurons diminishes the effects of SDF1 on these cells in culture and the effects of a conditioning lesion in vivo. Conversely, SDF1 enhances regeneration of the sciatic nerve and of ascending spinal sensory axons (Figure 9E). Strikingly, combining Ocm and SDF1 mimics or exceeds the effects of a conditioning lesion in accelerating both peripheral nerve and spinal cord axon regeneration [49]. Human monocytes express both Ocm and SDF1 (Figure 9F), and human sensory neurons generated from induced pluripotent stem cells (iPSCs) express both ArmC10 and CXCR4. Ocm promotes neurite outgrowth from human iPSC-derived sensory neurons (Figure 9F), a finding which suggests that the same ligand–receptor interactions that enable central and peripheral axon regeneration in mice may be of clinical benefit following neural injury in human patients [49].

## 10. Transcriptional Regulation of the Regenerative Program

**Figure 10:**
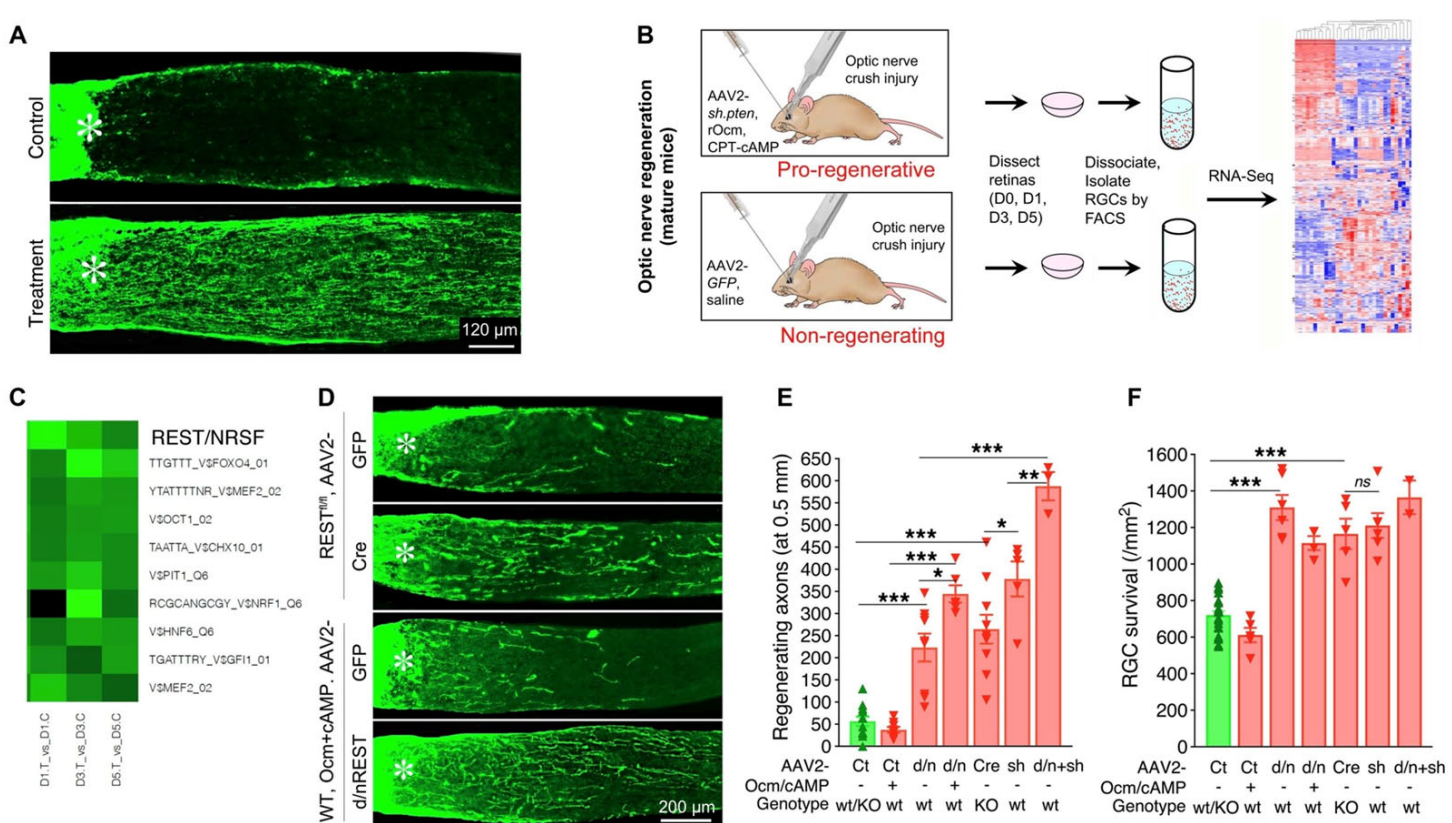
Transcriptional regulation of optic nerve regeneration. (**A**) longitudinal sections through the injured optic nerve immunostained for anterograde tracer CTB. Whereas a control treatment (AAV2-GFP + saline) stimulates almost no axon growth past the injury site, the combination of AAV2-*shPten* + Ocm + cAMP induces strong regeneration. (**B**) at early time points (1, 3, or 5 days) after optic nerve injury and treatment, retinas were dissected and dissociated. RGCs were isolated by fluorescence-activated cell sorting (FACS) and their transcriptomes analyzed by RNA Seq. Following identification of differentially expressed genes, bioinformatics was used to predict transcription factors that regulate the expression of genes that are either up- or down-regulated selectively in RGCs undergoing axon regeneration. (**C**) the transcription factor REST is predicted to be a strong negative regulator of genes associated with the regenerative. (**D**–**F**) using either *Rest*^flx/flx^ mice with intraocular injection of AAV2-Cre, or WT mice injected intraocularly with a virus expressing a dominant negative REST mutant (AAV2-d/nREST), REST suppression induced appreciable optic nerve regeneration and improved RGC survival. (**E**,**F**) when combined with Ocm+cAMP or with AAV2-shPten, REST KO further augments axon regeneration but not RGC survival. * *p* < 0.05; ** *p* < 0.01; *** *p* < 0.001 (panels reprinted with permission ref. [17]. Copyright 2022 Springer Nature).

As noted above, our earlier studies had shown that RGCs undergo profound transcriptional changes in response to intraocular inflammation, upregulating genes also known to be associated with peripheral nerve regeneration and the initial development of CNS axons [13]. Working with Drs. Daniel Geschwind, Riki Kawaguchi, and Giovanni Coppola (Functional Genomics Laboratory, UCLA), we combined in vivo treatments with RGC isolation via fluorescence-activated cell sorting (FACS), whole-transcriptome sequencing, and bioinformatics to predict the transcription factors that regulate the program of gene expression underlying optic nerve regeneration (Figure 10). A parallel study investigated the transcription factors that underlie that change in gene expression at the developmental time point at which RGCs lose their capacity to extend axons rapidly. Remarkably, the same transcription factors were predicted to be involved in both cases. Thus, an increase in the transcriptional repressor RE1-Silencing Transcription factor (REST, NRSF) was predicted to suppress the expression of growth-related genes postnatally, whereas REST inactivation was predicted to increase expression of genes that enable mature RGCs to regenerate their axons (Figure 10C). At the same time, the transcription factor Elk1 was predicted to be active in regulating gene expression during embryonic development, to be diminished or inactivated postnatally when RGCs lose their capacity for rapid axon growth, and to be reactivated when RGCs are stimulated to regenerate axons [97]. These predictions were borne out in gain- and loss-of-function studies. Deletion of the *Rest* gene in mature RGCs was sufficient to induce considerable axon regeneration, as was overexpression of a dominant negative form of REST that binds to appropriate DNA sequences but cannot engage the appropriate cofactors [17] (Figure 10D). Similarly, Elk1 overexpression was sufficient to increase axon regeneration and RGC survival after optic nerve injury [97]. Work from other laboratories has implicated multiple other transcription factors (e.g., c-myc, c-Jun, ATF3, KLF family members [98,99,100,101,102,103,104,105,106]), and epigenetic regulators (e.g., histone deacetylases [107,108,109,110,111,112]) in axon regeneration, and understanding the interactions among these will provide deeper insights into the gene regulatory networks underlying regeneration.

## 11. Role of Other Retinal Cell Types in Optic Nerve Regeneration

**Figure 11:**
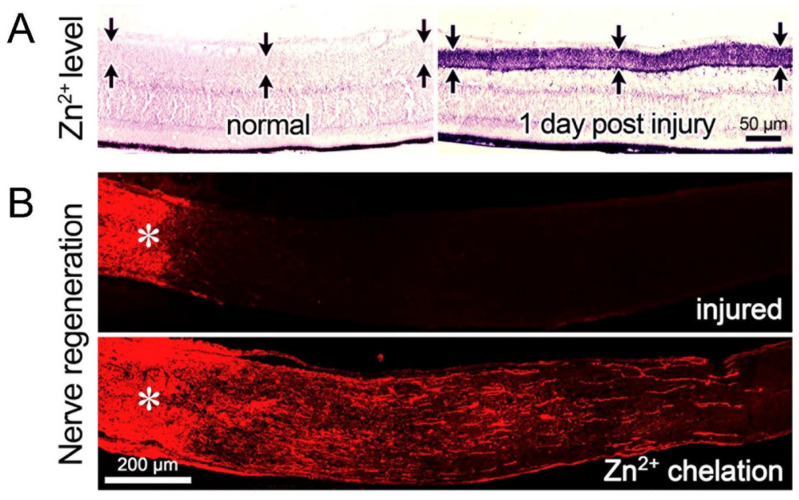
Accumulation of reactive zinc in amacrine cell terminals after optic nerve injury: another impediment to regeneration. (**A**) increase in autometallographic (AMG) signal presumed to reflect an elevation of reactive zinc (Zn^2+^) in synaptic terminals of amacrine cells in the inner plexiform layer (*arrows*) of the retina one day after optic nerve injury. (**B**) chelation of Zn^2+^ with ZX1 promotes axon regeneration beyond the site visualized two weeks after optic nerve injury. Asterisks denote the injury site (Reprinted with permission from ref. [116], copyright 2017, National Academy of Sciences).

RGCs’ ability to regenerate injured axons is also influenced by the other cells of the retina. In the early postnatal period, RGCs lose their intrinsic capacity to extend axons rapidly due to a contact-mediated signal from amacrine cells [113]. However, elevating Lin 28 expression in amacrine cells or blocking inhibitory neurotransmission from amacrine cells onto RGCs increases RGCs’ ability to regenerate axons in response to insulin-like growth factor-1 (IGF-1) [114]. It might be noted that others have reported the relevant loci of Lin 28 expression to be RGCs themselves [115]. RGCs’ survival and axon regeneration are also elevated by chelating zinc that accumulates in nerve terminals of amacrine cells shortly after optic nerve injury [116] (Figure 11), and ongoing work in our laboratory points to the role of nitric oxide produced by a subset of amacrine cells and dyshomeostasis of glutamate transport in bipolar cells in regulating amacrine cell Zn^2+^ levels and optic nerve regeneration (H.Y. Gilbert, Y.Q. Li, K. Omura, K. Yuki, L.I. Benowitz, and P.A. Rosenberg, in prep.). 

## 12. Regeneration and RGC Survival

Although RGCs’ ability to regenerate injured axons clearly depends on these cells’ survival, many studies point to divergent pathways in regulating survival and regeneration. For example, overexpressing the anti-apoptotic Bcl2 protein in mature RGCs is strongly neuroprotective after optic nerve injury but does not lead to appreciable axon regeneration [117]. Optic nerve injury leads to a rapid influx of Ca^2+^ at the injury site [118] and activation of the related protein kinases, DLK and LZK. Blocking these two kinases strongly increases the survival of injured RGCs but diminishes their capacity to regenerate axons in response to pro-regenerative treatment [119,120,121]. DLK/LZK activation in turn activates a downstream MAP kinase cascade that results in altered expression and/or activation of four transcription factors (c-Jun, ATF2, Mef2A, and SOX11), of which the contributions to survival and regeneration are currently under investigation. In contrast, another pathway involving germinal cell kinase four (GCK-IV) promotes both RGC survival and axon regeneration. GCK-IV inhibition is neuroprotective and enables some degree of regeneration on its own while also enhancing the pro-regenerative effects of *Pten* deletion [122]. These and related studies suggest that more treatments may be discovered that enable both robust RGC survival and robust axon regeneration. Another highly relevant discovery is that overexpression of a constitutively active form of the protein kinase calcium-calmodulin kinase IIα (CaMKIIα-T286D) is highly neuroprotective to RGCs in several conditions that would normally lead to cell death, including optic nerve crush, experimental glaucoma, and NMDA-mediated neurotoxicity [123]. Similar benefits, though of a somewhat lower magnitude, result from expressing an active form of the transcription factor CREB, a downstream target of CaMKII [123]. 

## 13. The Other Side of Inflammation

In contrast to the beneficial effects exerted by some immune cell-derived factors, there are other instances in which intraocular inflammation is highly deleterious to RGCs. Considerable evidence indicates that inflammation-derived molecules that include tumor necrosis factor-α (TNF-α) and the Fas ligand play an important role in the etiology of glaucoma [124,125,126,127,128,129,130,131,132,133,134,135,136], while other studies point to the role of TNF-α, interleukin 1-α (IL1α), and C1q in causing astrocytes to acquire neurotoxic properties and secrete long-chain saturated lipids that lead to RGC death [137,138]. In an animal model of Type I neurofibromatosis, heterozygous loss of the Nf1 gene coupled with a loss of heterozygosity in glial precursor cells results in the formation of non-cancerous tumors in the distal optic nerve and chiasm region that, in many cases, eventually causes RGCs to die [139,140,141,142,143,144,145]. In a prominent animal model of Type 1 neurofibromatosis, activation of haploinsufficient microglia by T-cell-derived CCL4 was found to contribute to disease progression by elevating CCL5, which enhances glioma growth. Thus, although there are clear benefits of controlled inflammation in promoting RGC neuroprotection and axon regeneration, inflammation under other conditions is highly toxic to RGCs. Discovering ways to modulate the immune response to favor a beneficial outcome is clearly a priority for the future. 

## 14. Conclusions

We have attempted to summarize what is known about the role of inflammation in promoting axon regeneration in the optic nerve, therapies that combine intraocular inflammation with other treatments (e.g., Pten deletion), and the role of non-cell autonomous factors such as the fibrotic scar, myelin-associated inhibitors, and other cells of the retina. Yet, despite the many advances to date in promoting optic nerve regeneration in animal models, the extent of regeneration achieved to date remains limited, underlining the need for greater efforts in this area. We do not yet know whether we will eventually be able to promote the long-distance regeneration of axons arising from many thousands of RGCs, or whether regenerating axons can form a topographic representation of the visual world in appropriate visual relay areas, both of which may be needed to restore image vision. And finally, it is essential to know whether what has been discovered in small animal models can carry over to non-human primates and eventually to patients. Much has been accomplished to date, but much remains to be done.
